# Aloe-emodin mediates the inhibitory effect of LncRNA D63785 on the PI3K/Akt/mTOR pathway in nasopharyngeal carcinoma

**DOI:** 10.3389/fphar.2025.1573408

**Published:** 2025-07-18

**Authors:** Min He, Lei Xie, Jiayi Huang, Han Su, Jiahua Hu, Liuping Xie, Mengqin Li, Xin Zeng, Jianhong Tang

**Affiliations:** ^1^ Department of Pharmacy, The Second Affiliated Hospital of Guilin Medical University, Guilin, China; ^2^ College of Pharmacy, Guilin Medical University, Guilin, China; ^3^ Guangxi Key Laboratory of Diabetic Systems Medicine, Guilin Medical University, Guilin, China; ^4^ Department of Traditional Chinese Medicine, The Second Affiliated Hospital of Guilin Medical University, Guilin, China; ^5^ Department of Neurology, The Affiliated Hospital of Guilin Medical University, Guilin, China; ^6^ Central Laboratory, Guangxi Health Commission Key Laboratory of Glucose and Lipid Metabolism Disorders, The Second Affiliated Hospital of Guilin Medical University, Guilin, China; ^7^ Guangxi Health Commission Key Laboratory of Glucose and Lipid Metabolism Disorders, The Second Affiliated Hospital of Guilin Medical University, Guilin, China; ^8^ School of General Medical, Guilin Medical University, Guilin, China

**Keywords:** AE, NPC, LncRNA D63785, PI3K/Akt/mTOR signaling pathway, proliferation and migration

## Abstract

**Background:**

Long non-coding RNAs (lncRNAs) are dysregulated in nasopharyngeal carcinoma (NPC), yet their interplay with pharmacological agents like aloe-emodin (AE) remains unclear. This study explores AE’s anti-NPC mechanisms via lncRNA D63785 and the PI3K/Akt/mTOR pathway.

**Methods:**

NPC cells (CNE1, C666-1) were treated with AE, followed by qRT-PCR and Western blotting to assess lncRNA D63785 and PI3K/Akt/mTOR pathway proteins. siRNA-mediated lncRNA D63785 knockdown combined with functional assays (CCK-8, EdU, colony/wound-healing) evaluated AE’s effects on proliferation, migration, and pathway activity. *In vivo* validation used nude mouse xenografts.

**Results:**

LncRNA D63785 was overexpressed in NPC cells (p < 0.01). AE suppressed lncRNA D63785 expression, concurrently reducing PI3K/Akt/mTOR phosphorylation (p < 0.05). siRNA knockdown partially reversed AE’s inhibition of NPC cell viability, proliferation, and migration. *In vivo*, AE attenuated tumor growth (p < 0.05), correlating with lncRNA D63785 downregulation and PI3K/Akt/mTOR dephosphorylation.

**Conclusion:**

AE exerts anti-NPC effects by targeting the lncRNA D63785-PI3K/Akt/mTOR axis, offering a novel therapeutic strategy. These findings bridge AE’s pharmacological activity with lncRNA regulatory networks in NPC pathogenesis.

## 1 Introduction

Nasopharyngeal carcinoma (NPC), a malignancy with distinct geographical prevalence in East and Southeast Asia, ranks as the third most common cancer in Southern China (Liu et al., 2021; Su et al., 2022). Its multifactorial etiology involves genetic susceptibility, Epstein-Barr virus (EBV) infection, and lifestyle factors such as nitrosamine-rich diets, smoking, and alcohol consumption (Chang et al., 2021; Jicman Stan et al., 2022). Despite advances in radiotherapy and chemoradiotherapy, clinical outcomes remain suboptimal, with a 5-year survival rate below 80% due to frequent late-stage diagnoses and treatment limitations like therapeutic resistance and adverse effects (Wu et al., 2016; Tang et al., 2021). These challenges underscore the urgent need for novel therapeutic strategies targeting NPC pathogenesis.

Natural compounds have emerged as promising candidates for anticancer drug development. Paclitaxel, evodiamine, and curcumin exemplify plant-derived agents with validated efficacy in oncology (Şeker Karatoprak et al., 2022; Abu Samaan et al., 2019; Luo et al., 2021; Shafei et al., 2021; Termini et al., 2020). Among these, aloe-emodin (AE), an anthraquinone isolated from Aloe vera, exhibits multifaceted pharmacological properties, including anti-inflammatory, immunomodulatory, and antitumor activities (Sanders et al., 2018). Preclinical studies highlight AE’s ability to suppress proliferation, migration, and invasion across diverse malignancies, such as cervical cancer (via suppression of HPV E6/E7 oncoproteins and GLUT1-mediated glucose metabolism (Gao et al., 2019), melanoma (through Wnt/β-catenin inhibition (Du et al., 2021)), colon cancer (via mitochondrial-mediated apoptotic pathways) (Jiang et al., 2021)), and breast cancer (by inhibiting telomerase activity (Wang S. et al., 2020)). Notably, AE’s antitumor effects in NPC remain underexplored, warranting mechanistic investigation.

The PI3K/Akt/mTOR pathway, a central regulator of cell survival, metabolism, and therapy resistance, is frequently dysregulated in NPC, driving tumor progression and chemoradiotherapy failure (Teng et al., 2021; Qin et al., 2020; Li et al., 2022; Chen et al., 2020). Hyperactivation of this pathway enhances NPC cell proliferation, invasion, and metastatic potential while conferring resistance to apoptosis (Xie et al., 2022; Zhang et al., 2020; Feng et al., 2019; Feng et al., 2023). Recent evidence implicates long non-coding RNAs (LncRNAs) as critical modulators of PI3K/Akt/mTOR signaling. For instance, LncRNA PTCSS1 promotes Akt phosphorylation to accelerate tumor growth in hepatocellular carcinoma (Sharma et al., 2022), whereas LncRNA MEG3 suppresses PI3K/Akt signaling in glioma (Jia and Yan, 2022). In NPC, aberrant LncRNA expression profiles correlate with malignant phenotypes, yet the functional roles of specific LncRNAs remain poorly characterized (Yao et al., 2022; Wang Y. et al., 2020; He et al., 2022).

Of particular interest is LncRNA D63785, a highly upregulated transcript in NPC tissues (Zheng et al., 2019). While its oncogenic role in gastric cancer involves miR-422a sequestration to induce chemotherapy resistance (Zhou et al., 2018), the mechanistic interplay between D63785 and PI3K/Akt/mTOR signaling in NPC remains uncharted. Building on AE’s documented PI3K/Akt inhibitory effects in other cancers (Peng et al., 2022; Zhu et al., 2023), we hypothesize that AE suppresses NPC progression by targeting LncRNA D63785 to attenuate PI3K/Akt/mTOR pathway activation.

Summary statement: In this study, we aim to explore the hypothesis that aloe-emodin inhibits NPC progression by targeting lncRNA D63785, thereby modulating the PI3K/Akt/mTOR signaling pathway. The results of this study may provide new insights into the therapeutic potential of aloe-emodin in NPC treatment.

## 2 Materials and methods

### 2.1 Materials

Human nasal mucosal epithelial cells (HNEpC, non-cancerous controls) were purchased from Guangzhou Suyan Biotechnology Co., Ltd. Nasopharyngeal carcinoma (NPC) cell lines CNE1, 5-8F, HONE1, and C666-1 were obtained from Xiangya Medical College of Central South University and Shanghai Yuchi Biotechnology Co., Ltd. Aloe emodin was purchased from Shanghai Yuanye Biotechnology. RPMI 1640 medium and EMEM medium were sourced from GIBCO and Guangzhou Suyan Biotechnology, respectively. Fetal bovine serum (FBS), penicillin-streptomycin (Cat# SV30010), TRIzol reagent (Cat# DP424), SYBR Green Master Mix, RIPA lysis buffer, BCA Protein Assay Kit, and Laemmli buffer were purchased from Sigma-Aldrich, Tiangen Biotech, Vazyme Biotech, and Beyotime Biotechnology. qPCR primers for LncRNA D63785 and GAPDH (internal control) were synthesized by Sangon Biotech (Shanghai, China). Opti-MEM, Lipofectamine 3000, and EdU reagent were obtained from Thermo Fisher Scientific. Primary antibodies against AKT, p-AKT, PI3K, p-PI3K, mTOR, p-mTOR, and β-actin were purchased from Affinity Biosciences, ABclonal Biotechnology, and Servicebio Biotechnology. HRP-conjugated goat anti-rabbit IgG secondary antibody and ECL Prime Western blotting Substrate were sourced from Beyotime Biotechnology. D63785-targeting siRNA (sense: 5′-GGC​AGU​UCC​ACA​GAA​UUU​TT-3′, antisense: 5′-AAA​UCU​GUG​GAA​UCT​CTT-3′; Cat# 338888) and negative control siRNA were synthesized by GenePharma. BALB/c nude mice (SPF-grade, Cat# SCXK 2019-0004) were purchased from SLAC Jingda Experimental Animal Co., Ltd. Isoflurane was obtained from Sigma-Aldrich. DMSO was purchased from Beijing Solarbio Science & Technology Co., Ltd. All reagents and antibodies were used in accordance with the manufacturers protocols.

### 2.2 Cell culture

NPC cells (CNE1, 5-8F, HONE1, C666-1) were cultured in RPMI 1640 medium supplemented with 10% FBS and 1% penicillin-streptomycin. HNEpC cells were maintained in EMEM medium with identical supplements. All cells were incubated at 37°C in 5% CO_2_.

Our previous studies have shown that AE inhibits the activity of CNE1 and C666-1 cells (Chen et al., 2023). A concentration of 20 μM was selected based on ∼70% cell viability after 48 h of treatment. For dose-response studies, concentrations were adjusted in 10 μM increments or decrements relative to 40 μM, which exhibited significant cytotoxicity.

### 2.3 Real time quantitative PCR

Total RNA was extracted from samples using TRIZOL reagent according to the manufacturer’s instructions. RNA concentration and purity were determined using the NanoDrop 2000 spectrophotometer. Reverse transcription was performed with 1 μg of RNA in a 20 μL reaction volume following the PrimeScript™ RT Master Mix protocol. Quantitative PCR amplification was carried out in triplicate using SYBR Green Master Mix under optimized cycling conditions: initial denaturation at 95°C for 30 s, followed by 40 cycles of 95°C for 10 s and 60°C for 30 s. Melt curve analysis confirmed amplification specificity. Relative gene expression was calculated using the 2^−ΔΔCT^ method with GAPDH normalization. The assay was performed three times independently.

### 2.4 Cell Western blot

Total protein was extracted from cells or tumor tissues using RIPA lysis buffer and denatured at 100°C for 10 min in Laemmli buffer. Protein concentrations were quantified via BCA assay, with 100 μg of total protein loaded per lane for separation on 10% SDS-PAGE gels. Electrophoresed proteins were transferred to PVDF membranes at 250 mA constant current for 90 min. Membranes were blocked with 5% non-fat milk (for non-phosphorylated targets) or 5% BSA (for phosphorylated proteins) for 2 h at room temperature. Primary antibodies diluted in TBST containing 5% BSA were incubated with membranes overnight at 4°C, followed by three 10-min TBST washes. Membranes were subsequently incubated with HRP-conjugated secondary antibody for 1 h at room temperature. Protein bands were visualized using ECL substrate and quantified through densitometric analysis. All the Western blot experiments were repeated at least three times.

### 2.5 Transfection

For siRNA transfection, 125 μL of Opti-MEM was mixed with 100 pmol siRNA in a 1.5 mL tube, followed by the addition of 4 μL LipoRNAi™ Transfection Reagent. The mixture was vortexed briefly and incubated at room temperature for 20 min to form siRNA-lipid complexes. NPC cells in logarithmic growth phase were seeded into 6-well plates at a density of 2 × 105 cells/well and cultured until 70%–80% confluency. The siRNA-lipid complexes were then added to cells and incubated for 48 h under standard culture conditions. Transfection efficiency was verified through quantitative PCR analysis prior to functional experiments. All procedures were performed in triplicate to ensure experimental consistency.

### 2.6 CCK-8

CNE1 and C666-1 cells were seeded into 96-well plates at a density of 1 × 10^4^ cells/mL (100 μL/well) and allowed to adhere for 24 h. Five experimental groups were established: (1) untreated control, (2) negative control (NC) with empty plasmid transfection, (3) D63785-targeting siRNA transfection, (4) 20μMAE treatment, and (5) combination therapy (siRNA+20μMAE). Following 48 h of treatment, 10 μL of CCK-8 reagent was added to each well and incubated in darkness at 37°C for 1.5 h. Optical density values were measured at 450 nm wavelength, with five technical replicates per group and three independent biological replicates. Data normalization and statistical analysis were performed relative to untreated controls.

### 2.7 Colony formation experiment

The colony formation assay was performed to evaluate proliferative capacity across experimental groups: (1) untreated control, (2) negative control (empty plasmid transfection), (3) D63785-targeting siRNA transfection, (4) 20μMAE treatment, and (5) combination therapy (siRNA+20μMAE). CNE1 and C666-1 cells in logarithmic growth phase were seeded at 200 cells/well in 6-well plates (three wells per group) and allowed to adhere for 24 h. After 48 h of treatment, the medium was replaced with fresh complete medium, and cells were cultured for 10 days under standard conditions (37°C, 5% CO_2_). Colonies were fixed with 4% paraformaldehyde (4°C, 1 h), stained with 0.1% crystal violet for 15 min, and gently rinsed with distilled water. Colonies containing ≥50 cells were counted using ImageJ software. Three independent biological replicates were performed. Colony formation rate = number of colonies/number of inoculated cells ×100%.

### 2.8 EdU doping experiment

The EdU incorporation assay was conducted to assess cell proliferation across five experimental groups: (1) untreated control, (2) negative control (empty plasmid transfection), (3) D63785-targeting siRNA transfection, (4) 20 μM AE treatment, and (5) combination therapy (siRNA+20μMAE). CNE1 and C666-1 cells in logarithmic growth phase were seeded into 24-well plates at 1 × 10^4^ cells/well (three technical replicates per group) and allowed to adhere for 24 h. After 48 h of treatment, cells were incubated with 10 μM EdU working solution for 2 h at 37°C. Subsequent fixation with 4% paraformaldehyde (15 min, room temperature) and permeabilization with 0.3% Triton X-100 (15 min) preceded the Click reaction (100 μL/well, 30 min, darkness). Nuclei were counterstained with Hoechst 33,342 (1:1,000 in PBS) for 5 min. Fluorescence images were acquired using standardized exposure parameters across all groups. EdU-positive cells (red fluorescence) and total nuclei (blue fluorescence) were quantified using ImageJ 1.53t across five random fields per well. Three independent biological replicates were analyzed.

### 2.9 Scratch healing experiment

The wound healing assay was conducted to assess cell migration across five experimental groups: (1) untreated control, (2) negative control (empty plasmid transfection), (3) D63785-targeting siRNA transfection, (4) 20μMAE treatment, and (5) combination therapy (siRNA+20μMAE) NPC cells in logarithmic growth phase were seeded into 6-well plates at 2 × 105 cells/well (three technical replicates per group) and cultured until reaching 80% confluence. A sterile 200 μL pipette tip was used to create a uniform linear scratch in the cell monolayer. After washing three times with PBS to remove cellular debris, the control group received serum-free medium containing 1 × 10^−3^ μM DMSO (vehicle control),while the experimental group was added with serum-free medium containing different concentrations of AE. Scratch widths were recorded at 0 h (baseline) and 48 h using an Olympus IX83 inverted microscope (×10 objective) under phase-contrast mode. Five random fields per well were imaged with consistent illumination settings. Migration rates were quantified using ImageJ with the MRI Wound Healing Tool plugin by calculating:Scratch closure rate=(scratch width from 0 h to 48 h)/scratch width from 0 h multiplied by 100%.Three independent biological replicates were performed. All procedures were conducted under standard culture conditions (37°C, 5% CO_2_).

### 2.10 Cultivate nude mouse NPC subcutaneous transplant tumor model

Sixteen 4-week-old nude mice (18–20 g) were housed under standardized conditions (22°C–24°C, 12-h light/dark cycle) with autoclaved water and irradiated feed. Following a 7-day acclimatization period, NPC cells (1 × 10^7^ cells/200 μL PBS) were subcutaneously injected into the right upper lumbar region. Tumor volumes were calculated daily using the formula (V = ab2/2), where (a) and (b) represent the longest and shortest diameters, respectively. Mice were randomized into two groups (n = 8/group) upon reaching tumor volumes of ∼80 mm^3^. The treatment group received daily intraperitoneal injections of 20 mg/kg (Dou et al., 2019) aloe emodin in 5% DMSO/saline (200 μL/mouse), while controls received vehicle alone. Humane endpoints included tumor burden >2,000 mm^3^, >20% body weight loss, or impaired mobility. On day 10, mice were anesthetized with isoflurane, euthanized via cervical dislocation, and tumors were excised, weighed, and snap-frozen for molecular analysis. All animal experiments were conducted in accordance with the animal ethics of the Animal Research Center of Guilin Medical College (approval number: GLMC-IACUC-2022015).

### 2.11 Statistical analysis

All experiments were independently repeated three times (biological replicates) with three technical replicates each. Data were expressed as mean ± standard deviation (SD). For comparisons between two groups, Student’s t-test was performed. One-way ANOVA with Tukey’s post hoc test was applied for multiple group comparisons. Statistical analyses were conducted using GraphPad Prism 8.0 (GraphPad Software). A probability value of P < 0.05 was considered statistically significant.

## 3 Results

### 3.1 AE reduced the expression of LncRNA D63785 and inhibited phosphorylation of PI3K/Akt/mTOR pathway in CNE1 and C666-1 NPC cells in a concentration-dependent manner

**FIGURE 1:**
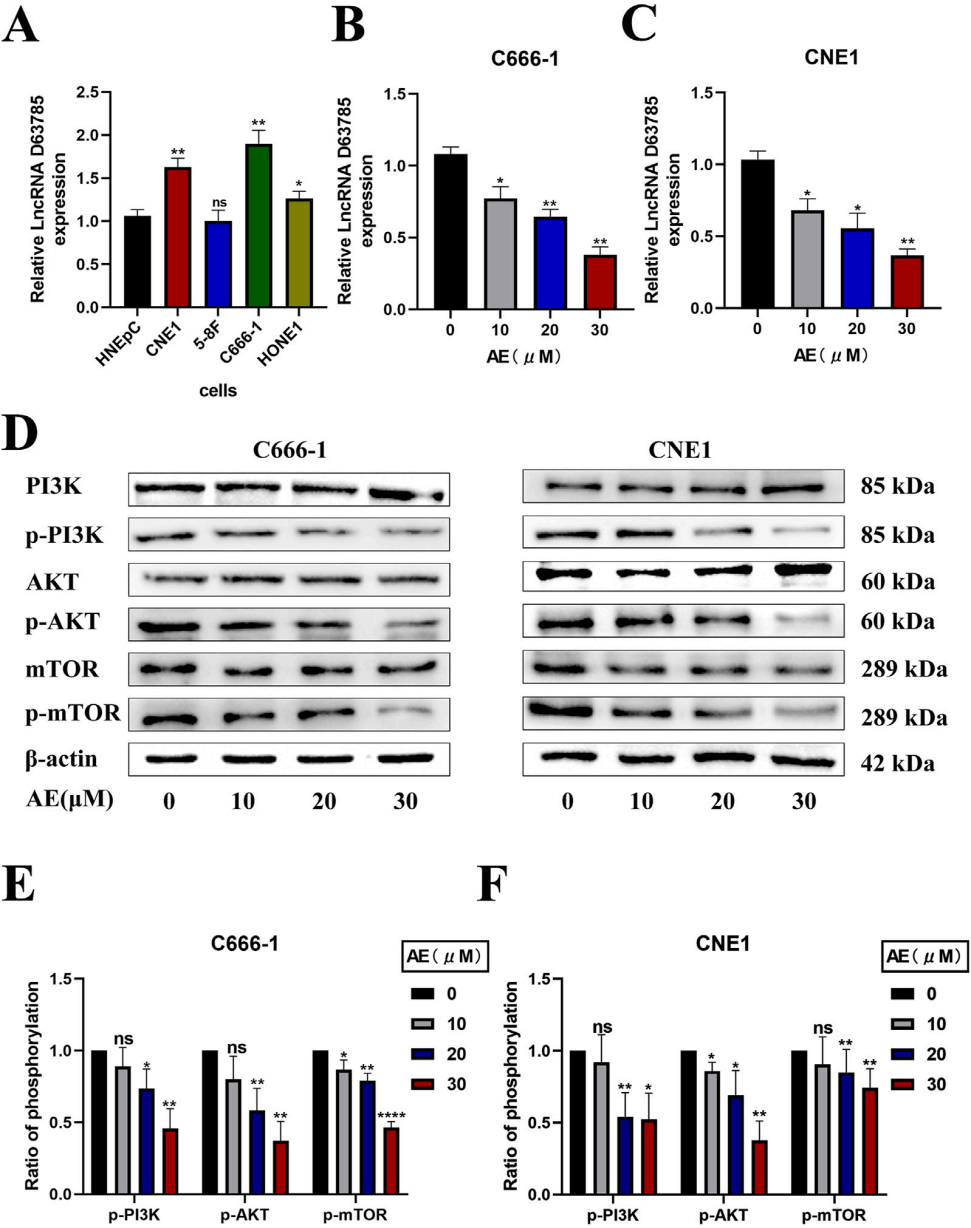
AE downregulated lncRNA D63785 and inhibited the PI3K/Akt/mTOR pathway in CNE1 and C666-1 NPC cells in a concentration-dependent manner. AE significantly inhibits the expression of lncRNA D63785 in NPC cells, and this effect is enhanced with increasing concentrations. **(A)** Expression of LncRNA D63785 in normal nasal mucosal epithelial cells and multiple nasopharyngeal carcinoma cells. Comparison of LncRNA D63785 self-expression between normal human nasal mucosal epithelial cells HNEpC and nasopharyngeal carcinoma CNE1, 5-8F, C666-1, HONE1 cells. The data shown is mean ± standard deviation, n = 3. ^ns^P>0.05,*P < 0.05, **P < 0.01, compared to the HNEpC group. **(B,C)** Changes in LncRNA D63785 under the action of aloe-emodin. The control group and different concentration gradients of aloe-emodin were treated on C666-1 cells for 48 h, and the expression of LncRNA D63785 was detected by real-time quantitative PCR **(B)**. The expression of LncRNA D63785 was detected by real-time quantitative PCR in CNE1 cells treated with control group and different concentration gradients of aloe-emodin for 48 h **(C)**. The data shown is mean ± standard deviation, n = 3. *P < 0.05, **P < 0.01, compared to the control (0) group. **(D–F)** The effect of aloe-emodin on the PI3K/Akt/mTOR signaling pathway in nasopharyngeal carcinoma cells. **(D)** The expression of pathway proteins in C666-1 and CNE1 cells was detected by Western blot after 48 h of treatment with control group and different concentration gradients of aloe-emodin. **(E,F)** Bar chart of phosphorylation protein expression of C666-1 and CNE1 cells under the action of control group and different concentration gradients of aloe-emodin. The data shown is mean ± standard deviation, n = 3. ^ns^P>0.05,*P < 0.05, **P < 0.01, compared to the control (0) group.

Real-time quantitative PCR analysis of LncRNA D63785 expression in normal nasal mucosal epithelial HNEpC cells and NPC cell lines (CNE1, 5-8F, C666-1, HONE1) revealed elevated LncRNA D63785 levels in CNE1, C666-1, and HONE1 cells compared to HNEpC controls (Figure 1A). The 5-8F cell line showed comparable expression to HNEpC cells. CNE1 and C666-1 cells, which exhibited the highest LncRNA D63785 expression, were further analyzed.

Treatment of CNE1 and C666-1 cells with AE (10, 20, 30 μM) for 48 h resulted in progressive reductions in LncRNA D63785 expression levels, as quantified by qPCR (Figures 1B,C).

Western blot analysis demonstrated that AE treatment (10, 20, 30 μM) did not alter total PI3K, Akt, or mTOR protein levels in either cell line (Figures 1D–F). However, phosphorylated forms of these proteins (p-PI3K, p-Akt, p-mTOR) showed concentration-dependent decreases in both C666-1 (Figures 1D,E) and CNE1 cells (Figures 1D,F).

### 3.2 LncRNA D63785 may mediate AE-inhibited NPC cell viability

**FIGURE 2:**
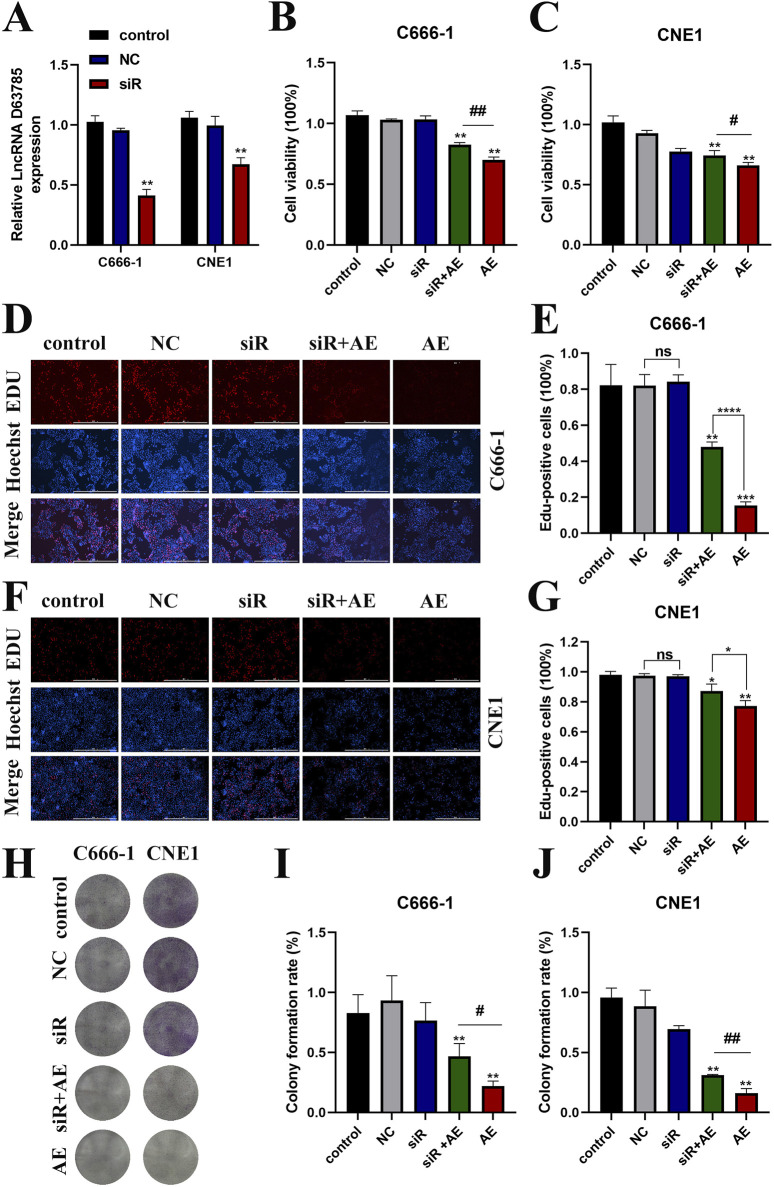
AE inhibited NPC cell vibility may be mediated by LncRNA D63785. **(A)** Expression of LncRNA D63785 in nasopharyngeal carcinoma cells transfected with siRNA. The control group, transfected empty plasmid group, and transfected siRNA group were treated with nasopharyngeal carcinoma C666-1 and CNE1 cells for 48 h, and the expression of LncRNA D63785 was detected by real-time quantitative PCR. The data shown is mean ± standard deviation, n = 3. *P < 0.05, **P < 0.01, compared to the control group. **(B,C)** Knockdown of LncRNA D63785 in NPC cells inhibits cell activity. The control group, empty group (NC) siRNA group, AE group, and siRNA + AE group were treated for 48 h, and the viability of C666-1 **(B)** and CNE1 **(C)** was detected by CCK-8. The data shown is mean ± standard deviation, n = 3. *P < 0.05, **P < 0.01, compared to the control group; ^#^P < 0.05,^##^P < 0.01, compared to the siRNA + AE group. **(D–G)** The control group, empty group (NC), siRNA group, AE group, and siRNA + AE group were treated for 48 h, and the proliferation rate of nasopharyngeal carcinoma cells was detected by EdU incorporation experiment. **(H–J)** The control group, empty group (NC), siRNA group, AE group, and siRNA + AE group were treated for 48 h, and the proliferation rate of nasopharyngeal carcinoma cells was detected through colony formation assay. Bar = 100 μm. The data shown is mean ± standard deviation, n = 3. *P < 0.05, **P < 0.01, compared to the control group; ^#^P < 0.05, ^##^P < 0.01, compared to the siRNA + AE group.

LncRNA D63785 knockdown models were established in CNE1 and C666-1 cells through siRNA transfection. qRT-PCR confirmed reduced LncRNA D63785 expression in siRNA-transfected cells compared to siNC controls (Figure 2A).

CCK-8 assays revealed decreased cell viability in AE-treated groups relative to untreated controls. This reduction was attenuated in cells co-treated with siRNA D63785 and AE (Figures 2B,C).

Colony formation assays demonstrated fewer colonies in AE-treated cells compared to controls. Co-treatment with siRNA D63785 and AE resulted in increased colony formation relative to AE treatment alone (Figures 2D–G).

EdU incorporation assays showed reduced proliferation rates in AE-treated groups. Co-treatment with siRNA D63785 and AE led to higher proliferation rates compared to AE-only groups (Figures 2H–J).

### 3.3 AE reduced the migration ability of NPC cells partly by affecting LncRNA

**FIGURE 3:**
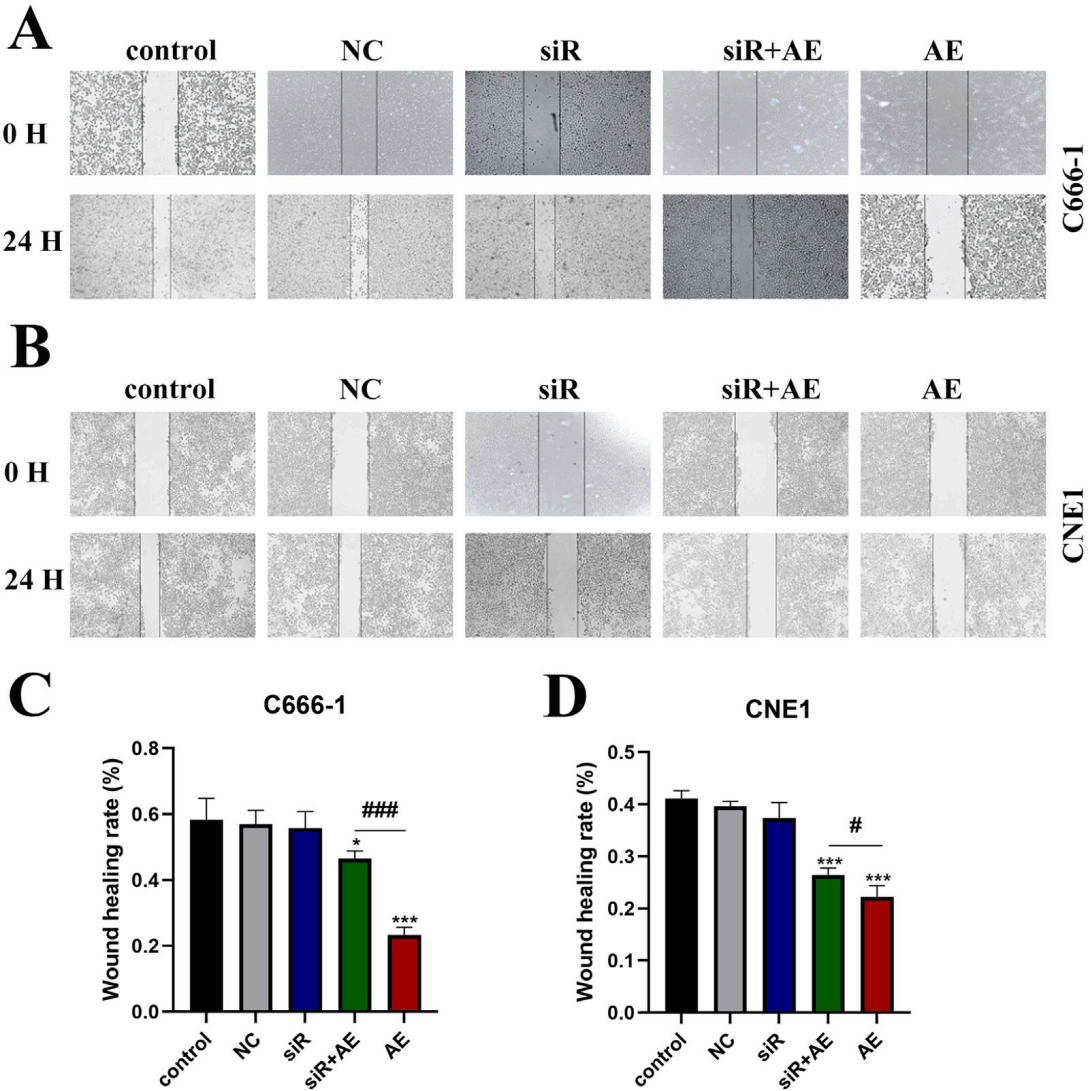
AE exerted its inhibitory effect on NPC cell migration ability through LncRNA D63785. **(A,B)** The control group, empty group (NC) siRNA group, AE group, and siRNA + AE group were treated for 48 h, and the scratch healing experiment was used to detect the migration ability of nasopharyngeal carcinoma cells C666-1 **(A)** and CNE1 **(B)**. Bar = 100 μm. **(C,D)** Histogram of migration ability of C666-1 **(C)** and CNE1 **(D)** cells. The data shown is mean ± standard deviation, n = 3. *P < 0.05, *P < 0.01, compared to the control group; ^#^P < 0.05, ^##^P < 0.01, compared to the siRNA + AE group.

Wound-healing assays were performed to assess NPC cell migration following treatments. Compared to untreated controls, AE-treated groups exhibited reduced scratch closure rates in both C666-1 (Figures 3A–C) and CNE1 (Figures 3B,D) cell lines. Transfection with empty vector (NC) or siRNA D63785 alone showed no measurable impact on migration compared to controls. In AE-treated cells, concurrent siRNA D63785 transfection attenuated the migration-inhibitory effects observed in AE-only groups.

### 3.4 The effect of knocking down LncRNA D63785 on PI3K/Akt/mTOR pathway proteins

**FIGURE 4:**
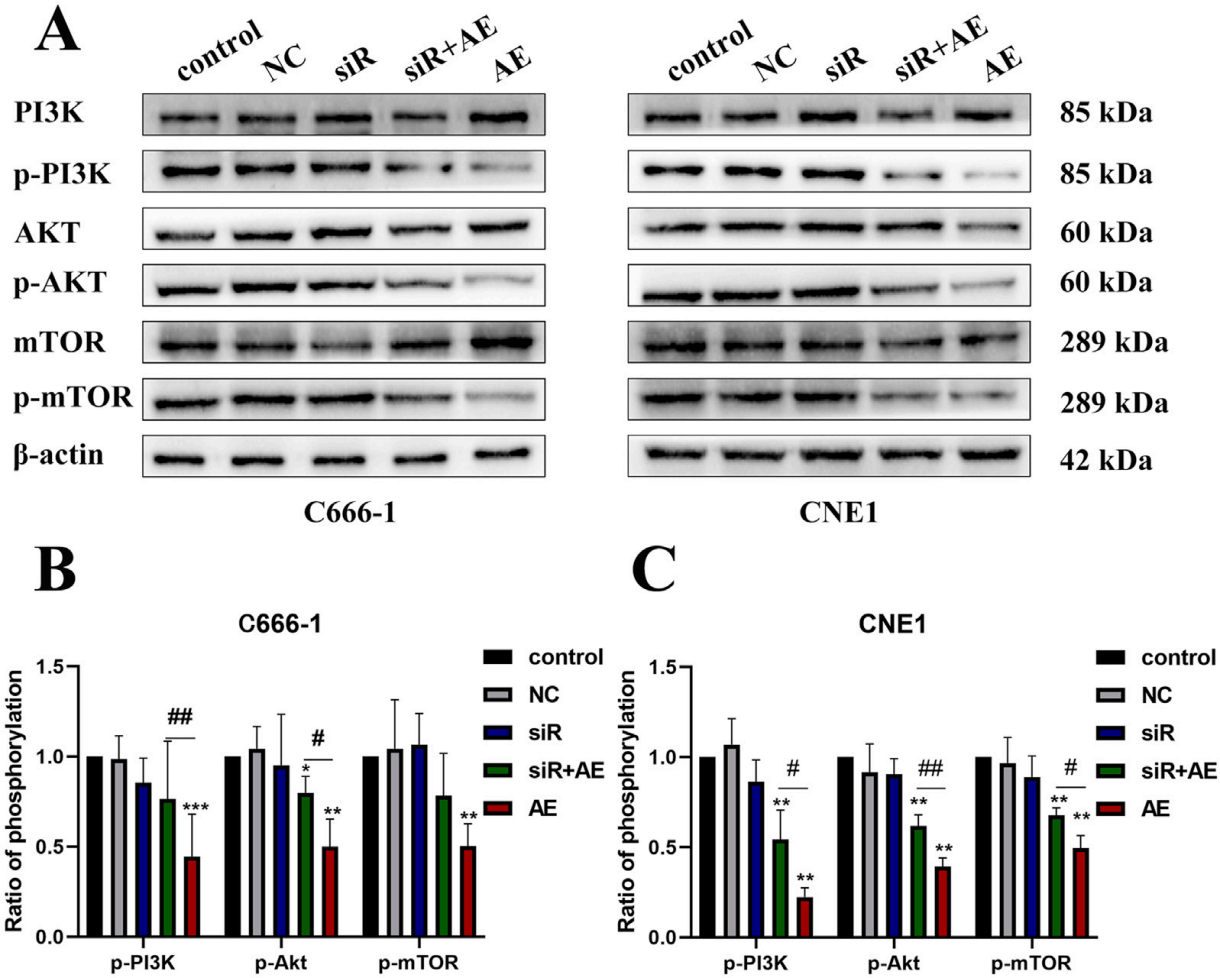
The effects of aloe-emodin and knocking down LncRNA D63785 on the PI3K/Akt/mTOR signaling pathway in nasopharyngeal carcinoma cells. **(A)** The control group, empty group (NC) siRNA group, AE group, and siRNA + AE group were treated for 48 h, and the expression of pathway proteins was detected by Western blot. Histogram of C666-1 **(B)** and CNE1 **(C)** cells phosphorylated proteins p-PI3K, p-Akt, and p-mTOR expression. The data shown is mean ± standard deviation, n = 3. ^ns^P>0.05, *P < 0.05, **P < 0.01, compared to the control group; ^#^P < 0.05, ^##^P < 0.01, compared to the siRNA + AE group.

Western blot analysis evaluated PI3K/Akt/mTOR pathway protein expression in C666-1 and CNE1 cells under five experimental conditions: control, empty vector (NC), siRNA D63785, AE (20 μM), and siRNA + AE co-treatment (Figure 4A). β-actin served as the loading control for protein normalization.

Compared to control groups, AE treatment reduced phosphorylated PI3K (p-PI3K), Akt (p-Akt), and mTOR (p-mTOR) levels in both cell lines. siRNA D63785 transfection alone did not alter baseline phosphorylation status relative to NC groups. In AE-treated cells, concurrent siRNA D63785 transfection attenuated AE-induced reductions in p-PI3K, p-Akt, and p-mTOR expression (Figures 4A–C).

### 3.5 AE inhibited the expression of LncRNA D63785 and the phosphorylation of PI3K/Akt/mTOR pathway proteins in an NPC tumor mouse model

**FIGURE 5:**
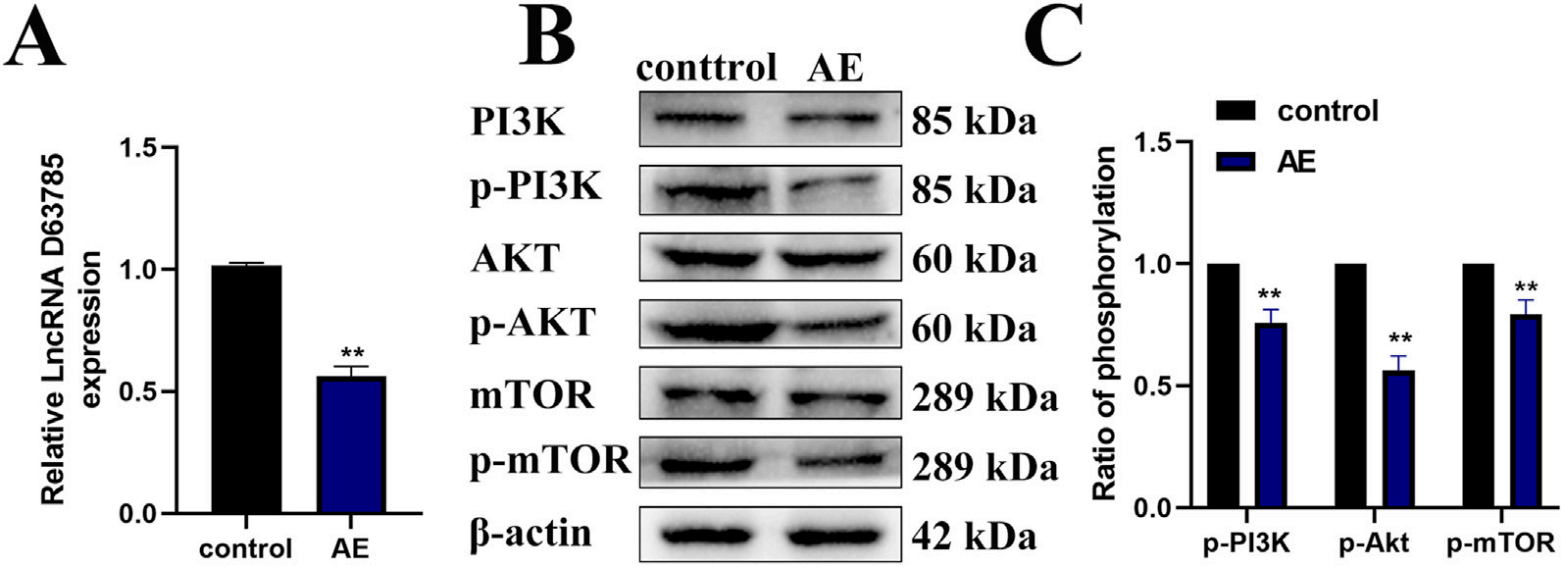
AE inhibits the expression of LncRNA D63785 and phosphorylation of PI3K/Akt/mTOR pathway proteins in subcutaneous transplanted tumors of NPC in nude mice. **(A)** Expression of LncRNA D63785 in the control group and aloe-emodin group. The data shown is mean ± standard deviation, n = 3, **P < 0.01, compared to the control group. **(B,C)** Expression of PI3K/Akt/mTOR signaling pathway proteins in the control group and aloe-emodin group. The data shown is mean ± standard deviation, n = 3, **P < 0.01, compared to the control group.

Our previous studies have shown that AE can inhibit tumor growth in NPC cells xenograft mouse models (Chen et al., 2023). Real-time quantitative PCR analysis of subcutaneous xenograft tissues revealed reduced LncRNA D63785 expression in the AE-treated group compared to controls (Figure 5A). Western blot analysis of the same tissues demonstrated decreased phosphorylation levels of PI3K/Akt/mTOR pathway proteins (p-PI3K, p-Akt, p-mTOR) in the AE-treated group relative to controls (Figures 5B,C). β-actin served as the loading control for protein normalization.

## 4 Discussion

As a head and neck cancer, distant metastasis and local recurrence are still the two main failure modes for NPC patients (Liu et al., 2021). After recurrence or metastasis, the treatment effect is poor, the prognosis is poor, and the survival rate is significantly reduced. Therefore, it is crucial to study the pathogenesis of NPC, search for new therapeutic targets, and find effective and low-toxicity anti-tumor drugs.

Aloe vera is a perennial herbaceous plant in the lily family, and it has many types, but Curacao aloe vera has been studied the most extensively. It is considered a “healing” plant with medicinal value and has been used for over 3,000 years in different cultures of many countries. AE has anti-tumor and anti-proliferative effects on various types of cancer and cell lines, like HeLa Cells (Gao et al., 2019). However, the molecular mechanism of NPC pathogenesis is still not fully understood, and research on how AE affects NPC is limited. This study, based on our research group’s previous research foundation, aims to elucidate the inhibitory effect of aloe-emodin on NPC.

LncRNA has excellent potential in cancer treatment and deserves more attention (Huang et al., 2025; Hsu et al., 2025). Previous reports lncRNAs play important roles in the occurrence and development of NPC, such as HOXA-AS2, HOTAIR, FOXD1-AS1, LINC00669, and so on (Wang et al., 2022). Moreover, LncRNA D63785 is highly expressed in NPC patient tissues (Zheng et al., 2019). Our result showed that LncRNA D63785 is highly expressed in multiple NPC cells as CNE1, C666-1, and HONE1 cell lines, but, interestingly, aloe emodin lacks inhibition of D63785 in 5–8F cells, indicating that cell environment dependence deserves further investigation. Moreover, we found that AE dose-dependently reduced the expression of D63785 in CNE1 and C666-1 cells, which is related to the decreased phosphorylation of PI3K/Akt/mTOR pathway proteins. Importantly, knocking down LncRNA D63785 alone does not significantly affect NPC cells. However, siRNA-mediated knockout of D63785 partially reversed the anti-tumor effects of aloe-emodin on cell survival, proliferation, migration, and PI3K/Akt/mTOR signaling, strongly indicating that D63785 downregulation mediates at least partial pharmacological activity of aloe-emodin in these models. Suggested LncRNA D63785 mediated AE-inhibited NPC cell viability, etc. The consistency of these findings *in vivo* that aloe-emodin reduces the expression of D63785 and the activation of PI3K/Akt/mTOR while inhibiting tumor growth-further supports this relationship. Moreover, the PI3K/Akt/mTOR signaling pathway is involved in the occurrence and development of tumors and is closely related to the clinical and pathological characteristics of NPC (Li et al., 2022), and its transmission is also of great significance for treating NPC (Zhang et al., 2019). Therefore, AE inhibits the proliferation of CNE1 and C666-1 cells by downregulating LncRNA D63785, and the PI3K/Akt/mTOR pathway may be involved. Although current evidence suggests that D63785 is a key downstream effector of aloe-emodin activity in NPC models with high D63785 expression, It has not yet been confirmed whether aloe emodin directly binds to D63785 RNA to make it unstable or indirectly acts through epigenetic modification factors/transcription factors that control D63785 transcription.

Previous studies have shown, upregulation of lncRNA HAGLROS enhances nasopharyngeal carcinoma development by modulating PI3K/AKT/mTOR signaling mediated by miR-100/ATG14 axis (Zhang et al., 2019). But our current research results indicate that D63785 knockout reduces the phosphorylation levels of PI3K, Akt, and mTOR without significantly altering their total protein expression, suggesting that its regulatory role lies in pathway activation rather than transcriptional control of core components. We hypothesize two possible mechanisms based on observed signal dynamics: 1) D63785 can stabilize pathway activation by interacting with phosphatases or kinases that regulate phosphorylation status, or 2) it can serve as a scaffold to facilitate signal complex assembly. The partial reversal of the knockout effect of aloe-emodin on LncRNA D63785 further supports its upstream regulatory position. It is necessary to study the interaction group of LncRNA D63785, and in the future, we will evaluate its impact on post-translational modifications.

In clinical practice, we discussed the potential use of D63785 as a biomarker for predicting sensitivity to aloe emodin, particularly in tumors with high baseline expression of D63785. We explored strategies for combining aloe emodin with PI3K inhibitors or conventional therapies to overcome drug resistance. In addition, the lack of patient-sourced data is a limitation, and we are collaborating with clinical institutions to validate our findings in patient tumor samples and correlate them with clinical outcomes in ongoing studies.

**FIGURE 6:**
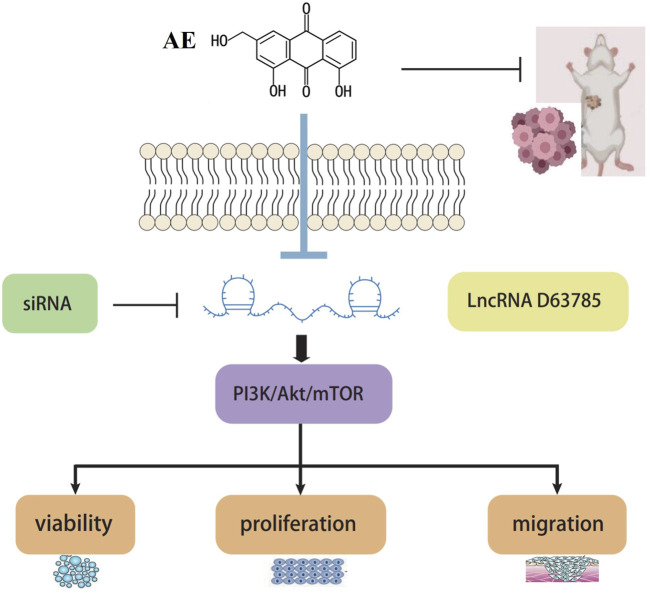
Graphical abstract.

In summary, this study found a novel target lncRNA D63785 with therapeutic potential, which regulates target proteins and affects NPC cell viability, elucidating an axis of AE targeting NPC and preliminarily elucidating the molecular mechanism of AE inhibition of NPC (Figure 6). It concludes that AE may mediate the PI3K/Akt/mTOR pathway regulation by LncRNA D63785, inhibiting the cell viability, proliferation, migration, and other malignant biological behaviors of NPC cells CNE1 and C666-1. AE may inhibit the growth of subcutaneous transplanted tumors in nude mice NPC through lncRNA D63785 and PI3K/Akt/mTOR pathway. This study starts with the search for long noncoding RNAs, regulates downstream proteins to affect NPC cell phenotype changes and tumor growth, elucidates a signaling pathway through which natural drugs act on tumors, and organically combines natural drug components, molecular mechanisms, cells, and tumor growth to provide ideas for the progress of NPC treatment. It is expected to explain the effects of natural drug components further and lay a solid foundation for later drug research and clinical applications.

## Data Availability

The original contributions presented in the study are included in the article/supplementary material, further inquiries can be directed to the corresponding author.
